# Long-Range Structural Order in a Hidden Phase of Ruddlesden–Popper Bilayer Nickelate La_3_Ni_2_O_7_

**DOI:** 10.1021/acs.inorgchem.3c04474

**Published:** 2024-03-05

**Authors:** Haozhe Wang, Long Chen, Aya Rutherford, Haidong Zhou, Weiwei Xie

**Affiliations:** † Department of Chemistry, Michigan State University, East Lansing, Michigan 48824, United States; ‡ Department of Physics and Astronomy, University of Tennessee, Knoxville, Tennessee 37996, United States

## Abstract

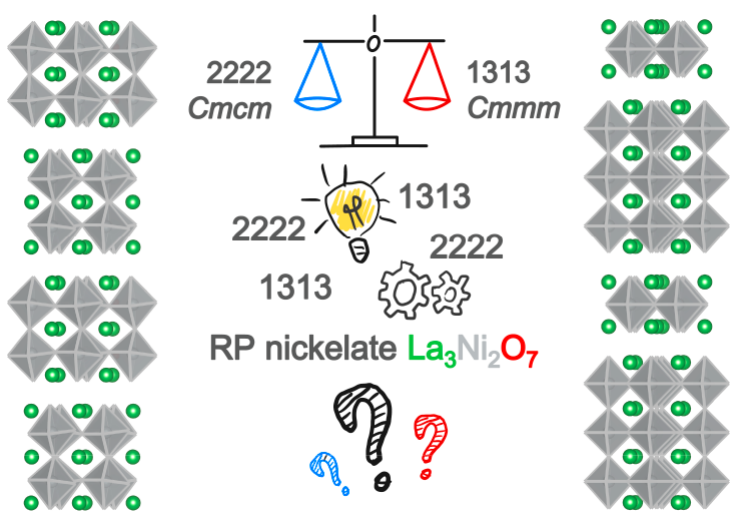

The recent discovery of superconductivity in the Ruddlesden–Popper bilayer nickelate, specifically La_3_Ni_2_O_7_, has generated significant interest in the exploration of high-temperature superconductivity within this material family. In this study, we present the crystallographic and electrical resistivity properties of two distinct Ruddlesden–Popper nickelates: the bilayer La_3_Ni_2_O_7_ (referred to as 2222-phase) and a previously uncharacterized phase, La_3_Ni_2_O_7_ (1313-phase). The 2222-phase is characterized by a pseudo *F*-centered orthorhombic lattice, featuring bilayer perovskite [LaNiO_3_] layers interspaced by rock salt [LaO] layers, forming a repeated ...2222... sequence. Intriguingly, the 1313-phase, which displays semiconducting properties, crystallizes in the *Cmmm* space group and exhibits a pronounced predilection for a *C*-centered orthorhombic lattice. Within this structure, the perovskite [LaNiO_3_] layers exhibit a distinctive long-range ordered arrangement, alternating between single- and trilayer configurations, resulting in a ...1313... sequence. This report contributes to novel insights into the crystallography and the structure–property relationship of Ruddlesden–Popper nickelates, paving the way for further investigations into their unique physical properties.

## Abstract 2

This study delves into the crystallographic and electrical properties of two polymorphs of Ruddlesden−Popper nickelates La_3_Ni_2_O_7_: bilayer 2222-phase and a hidden 1313-phase. The 1313-phase demonstrates a *C*-centered orthorhombic lattice (*Cmmm*, #65) with long-range ordering alternating between the single layer and trilayer (...1313... sequence). This unique layer stacking shed light on the structure−property relationship in La_3_Ni_2_O_7_, opening avenues for further exploration of their physical properties.

## Introduction

Nickelates have emerged as promising candidates for the exploration of additional high-temperature superconductors and for understanding the origins of high *T*_C_ superconductivity in cuprate superconductors reported in the 1980s.^1^ This interest stems from their analogous crystal and electronic structures.^2^ Two major families are Ruddlesden–Popper phases and reduced square-planar phases. Ruddlesden–Popper phases feature perovskite layers with octahedral coordination [NiO_6_] separated by rock salt layers, represented by the general formula *R*_*n*+1_Ni_*n*_O_3*n*+1_ (*n* = 1, 2, 3, ..., ∞).^3,4^ On the other hand, reduced square-planar phases exhibit square net layers with square-planar coordination [NiO_4_], where rock salt layers transform to fluorite blocking slabs, summarized by the general formula *R*_*n*+1_Ni_*n*_O_2*n*+2_ (*n* = 1, 2, 3, ..., ∞).^5−9^

Previously, superconductivity has been reported in epitaxial thin films of reduced square-planar phases, including infinite-layer Nd_0.8_Sr_0.2_NiO_2_,^10^ Pr_0.8_Sr_0.2_NiO_2_,^11,12^ La_1–*x*_Sr_*x*_NiO_2_,^13^ La_1–*x*_Ca_*x*_NiO_2_,^14^ and quintuple-layer Nd_6_Ni_5_O_12_.^15,16^ These systems feature ultralow valence Ni^1+^, isostructural to Cu^2+^, and the induction of hole-doping suggests that superconductivity stabilizes with a similar formal 3d electron count. However, the synthesis of these epitaxial thin films through topotactic reduction with metal hydrides introduces the possibility of hydrogen doping, casting ambiguity on the origin of superconductivity.

Recently, a groundbreaking discovery reported superconductivity in the Ruddlesden–Popper bilayer nickelate La_3_Ni_2_O_7−δ_, achieving a *T*_C_ up to 80 K in the pressure range of 14 to 43.5 GPa,^17^ sparking significant interest in Ruddlesden–Popper phases (*n* = 2 and *n* = 3). In the ambient pressure structure of the Ruddlesden–Popper bilayer La_3_Ni_2_O_7−δ_, the NiO_6_ octahedra display rotation/tilt alignment along the *c*-axis, deviating from the regular square net observed in high *T*_C_ cuprates. It has been proposed that a structural transition from ambient pressure *Amam* to the high-pressure *Fmmm* phase occurs at approximately 14 GPa, coinciding with the onset of superconductivity. However, the high-pressure phase remains unclear due to the resolution limitations of the reported powder X-ray diffraction (XRD) data. Additionally, the potential presence of oxygen vacancies introduces sample-dependent variability in multiple physical property measurements.^17−21^ The structural ambiguity and the crucial role of the structure–property relationship in comprehending the origins of high *T*_C_ superconductivity necessitate more focused attention and efforts in the study of the crystal structure in this system.

Herein, we present the crystal structure and electrical resistivity of two Ruddlesden–Popper nickelates: bilayer La_3_Ni_2_O_7_-2222, and a hidden phase, La_3_Ni_2_O_7_-1313. Single crystal XRD is employed to determine their crystal structures. Our measurements confirm the pseudo *F*-centered orthorhombic lattice and its Ruddlesden–Popper bilayer stacking in La_3_Ni_2_O_7_-2222. Remarkably, La_3_Ni_2_O_7_-1313 adopts a strongly preferred *C*-centered orthorhombic lattice with space group *Cmmm*. In this structure, perovskite [LaNiO_3_] layers exhibit a systematic long-range order, alternating between single- and trilayer configurations (...1313...). This report introduces new possibilities for exploring the crystal structure and the structure–property relationship in Ruddlesden–Popper nickelate superconductors.

## Experimental Section

### Materials Growth

Crystals of La_3_Ni_2_O_7_-2222 and La_3_Ni_2_O_7_-1313 were grown by a floating zone method at the University of Tennessee in a vertical optical-image furnace. Stoichiometric mixtures of La_2_O_3_ (pretreated at 1000 °C) and NiO were ground and fired at 1050 °C for 1 day. These precursor powders were hydrostatically pressed into a rod and sintered at 1400 °C for 12–24 h. Crystals of La_3_Ni_2_O_7_-2222 and La_3_Ni_2_O_7_-1313 were grown directly from the sintered rods in 100% O_2_ at a pressure of around 14–15 bar. During the crystal growth, the traveling rate was 3–4 mm/h, and the feed rod and seed were counter-rotated at a rate in the range of 15–20 rpm. La_3_Ni_2_O_7_-2222 and La_3_Ni_2_O_7_-1313 crystals were obtained from some sections of the grown boules. The two kinds of crystals were identified by single crystal X-ray diffraction. No uncommon hazards are noted in this experimental procedure.

### Single Crystal X-ray Diffraction Measurement

The single crystal of La_3_Ni_2_O_7_ was picked up, mounted on a nylon loop with paratone oil, and measured using a XtalLAB Synergy, Dualflex, Hypix single crystal X-ray diffractometer with an Oxford Cryosystems low-temperature device, operating at *T* = 300(1) K and *T* = 100(1) K. Data were measured using ω scans using Mo K_α_ radiation (λ = 0.71073 Å, microfocus sealed X-ray tube, 50 kV, 1 mA). The total number of runs and images was based on the strategy calculation from the program CrysAlisPro 1.171.43.92a (Rigaku OD, 2023). Data reduction was performed with correction for Lorentz polarization. Numerical absorption correction based on Gaussian integration over a multifaceted crystal model. Empirical absorption correction using spherical harmonics, implemented in SCALE3 ABSPACK scaling algorithm. The structure was solved and refined using the Bruker SHELXTL Software Package.^22,23^

### Electrical Resistivity Measurement

Temperature-dependent electrical resistivity measurement was performed with a Quantum Design DynaCool physical property measurement system (PPMS) in the temperature range 1.8–300 K at zero field with a four-probe method using platinum wires on a single crystal sample of La_3_Ni_2_O_7_-1313 in the dimensions of 3.5 mm × 2.8 mm × 1.2 mm.

## Results and Discussion

### Crystal Structure Determination

The crystal structures of Ruddlesden–Popper bilayer La_3_Ni_2_O_7−δ_ (δ = 0.00 and 0.08) were initially determined as *F*-centered orthorhombic *Fmmm* through powder X-ray diffraction (XRD) and Rietveld refinement.^24^ Recognizing the significant uncertainty in determining oxygen coordinates via XRD analysis, neutron powder diffraction (NPD) was subsequently employed.^25,26^ The results obtained indicated that the *Fmmm* space group was inappropriate as it failed to account for extra weak peaks, hinting at a lower symmetry. This led to the proposal that the most suitable structure model involves a *C*-centered orthorhombic lattice of the space group *Cmcm*.^26^ The challenges inherent in the pseudo *F*-centered orthorhombic lattice, the influence of oxygen vacancies on the structure, and the coexistence of Ruddlesden–Popper bilayer and trilayer phases underscore the complexities in structure determination, necessitating the growth of pure La_3_Ni_2_O_7_ single crystals. In a recent development, the high-pressure floating zone method, enabling 100% O_2_ atmosphere with controllable gas pressure, has successfully facilitated the single crystal growth of Ruddlesden–Popper nickelates with high purity.^27^ This advancement opens possibilities for studying crystal structures using X-rays, which are more cost-effective and accessible than neutrons and provide precise enough structure determination results.

**Figure 1:**
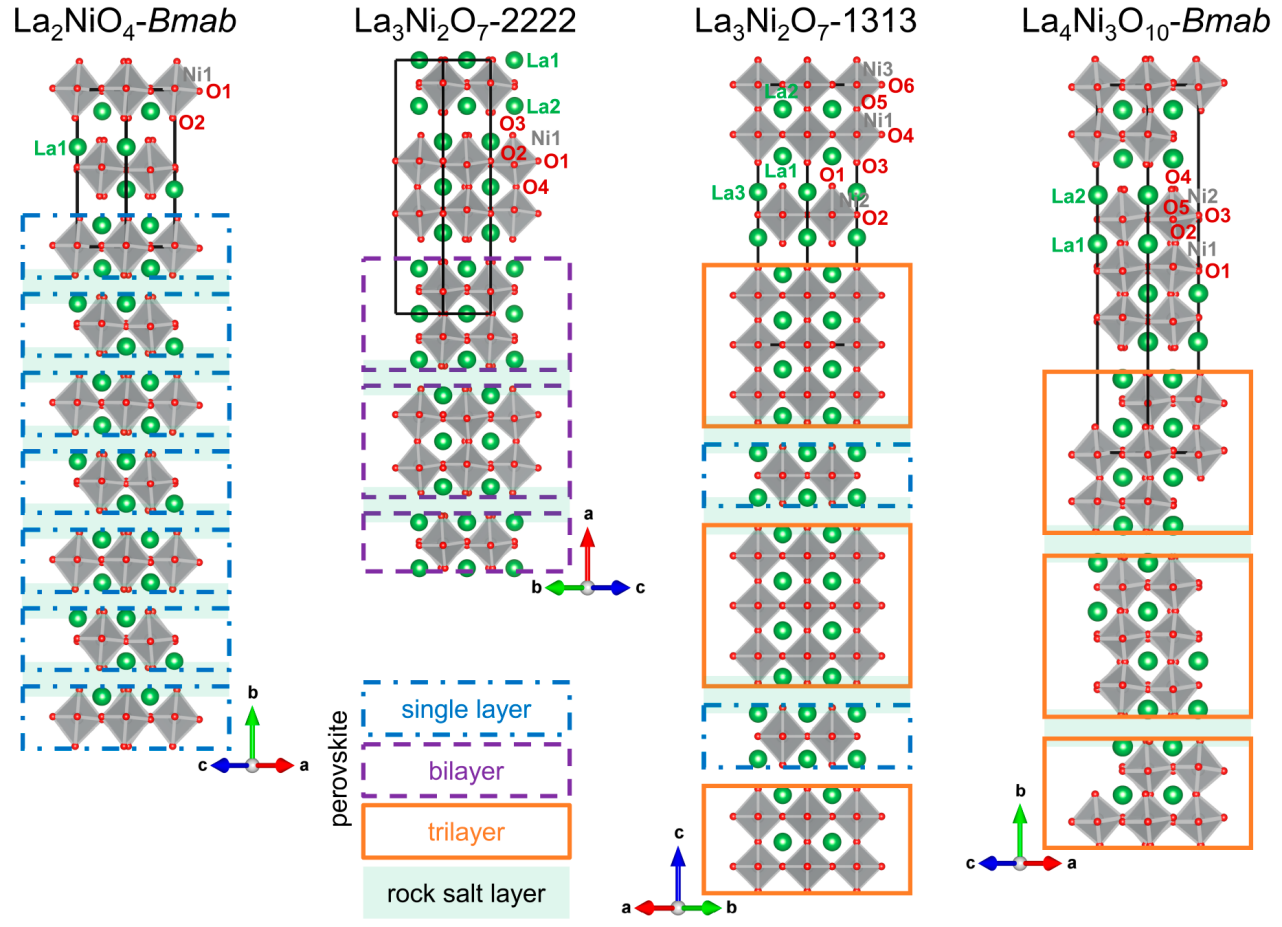
View of layer stacking in Ruddlesden–Popper nickelates, single layer La_2_NiO_4_, bilayer La_3_Ni_2_O_7_-2222, hidden phase La_3_Ni_2_O_7_-1313, and trilayer La_4_Ni_3_O_10_. Green, gray, and red represent La, Ni, and O atoms. Crystallographically unique atoms are labeled. Perovskite layers are highlighted with blue dashed-dotted lines, purple dashed lines, and orange lines, indicating *n* = 1, 2, and 3, respectively. Rock salt layers are colored with light green.

**Figure 2:**
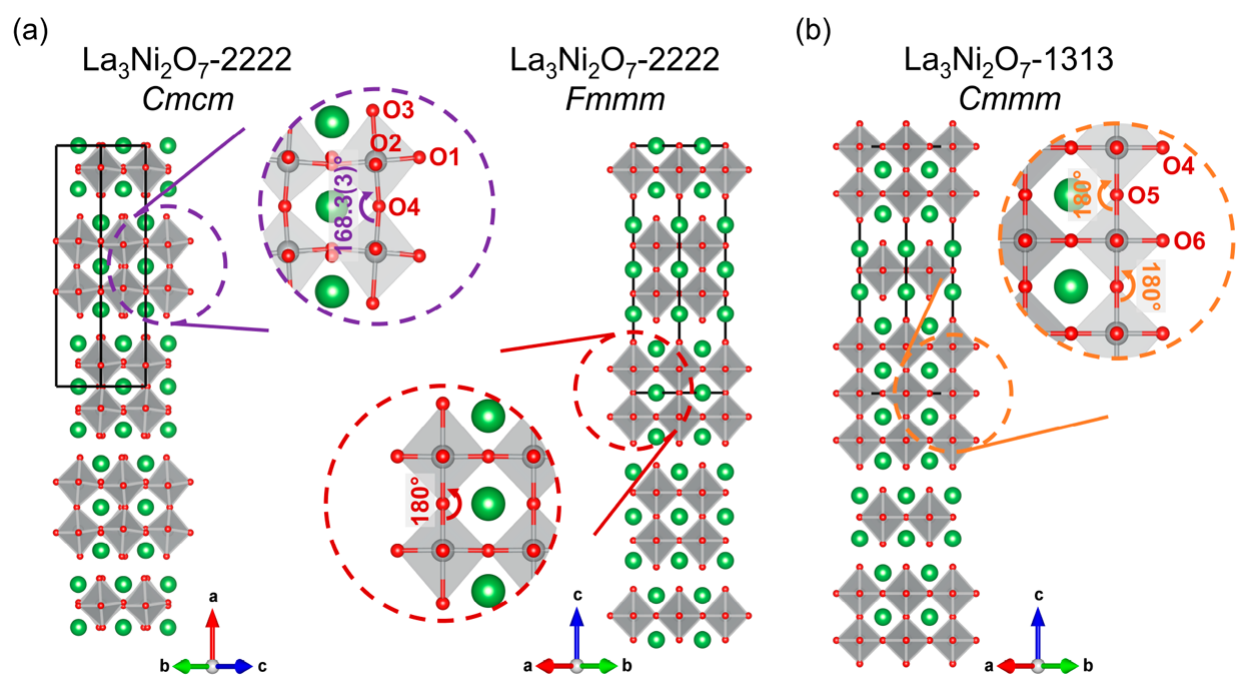
View of the characteristic out-of-plane Ni–O–Ni bond angle in (a) bilayer La_3_Ni_2_O_7_-2222 (*Cmcm* and *Fmmm* settings) and (b) hidden phase La_3_Ni_2_O_7_-1313. Green, gray, and red represent La, Ni, and O atoms. The corresponding oxygen atoms and bond angles are labeled as presented.

Here, utilizing this high-pressure floating zone method, high-purity single crystals of the Ruddlesden–Popper bilayer nickelate La_3_Ni_2_O_7_ (termed La_3_Ni_2_O_7_-2222) have been obtained. Our single crystal XRD investigations confirm the presence of a pseudo *F*-centered orthorhombic lattice and the characteristic Ruddlesden–Popper bilayer stacking, as shown in Figure 1. The single crystal XRD refinement details are summarized in Tables 1 and 2. No instances of oxygen vacancy have been observed with careful refinement attempts. The structural difference between the *F*-centered *Fmmm* and *C*-centered *Cmcm* models hinges on the positioning of oxygen atoms in the perovskite layers (labeled as O4 in La_3_Ni_2_O_7_-2222 in Figures 1 and a). Specifically, this distinction lies in whether oxygen resides on a symmetry-restricted special site (*Fmmm*) or a more general site (*Cmcm*). Of particular note the characteristic observed in the out-of-plane Ni–O–Ni bond angle emphasized in Figure 2. In the *Cmmm* configuration, this angle refines to 168.3(3)°, a significant deviation from the 180° observed in the *Fmmm* setting, providing additional evidence against the appropriateness of the *Fmmm* model. A recent report also suggests that this *C*-centered lattice becomes increasingly favorable as the temperature decreases.^28^

**Table 1:** Crystal Structure and Refinement of La_3_Ni_2_O_7_-2222 and La_3_Ni_2_O_7_-1313 at 300 K

chemical formula	La_3_Ni_2_O_7_-2222	La_3_Ni_2_O_7_-1313
formula weight	646.15 g/mol	646.15 g/mol
space group	*Cmcm*	*Cmmm*
unit cell dimensions	*a* = 20.5295(7) Å	*a* = 5.4399(2) Å
	*b* = 5.44607(17) Å	*b* = 5.4594(2) Å
	*c* = 5.3921(2) Å	*c* = 20.3265(5) Å
volume	602.86(4) Å^3^	603.66(4) Å^3^
*z*	4	4
density (calculated)	7.119 g/cm^3^	7.110 g/cm^3^
absorption coefficient	26.916 mm^–1^	26.881 mm^–1^
*F*(000)	1132	1132
2θ range	7.74 to 81.66°	6.02 to 82.62°
reflections collected	18169	19216
independent reflections	1059 [*R*_int_ = 0.0627]	1164 [*R*_int_ = 0.0755]
refinement method	full-matrix least-squares on *F*^2^	full-matrix least-squares on *F*^2^
data/restraints/parameters	1059/0/37	1164/0/49
final *R* indices	*R*_1_ (*I* > 2σ(*I*)) = 0.0267; *wR*_2_ (*I* > 2σ(*I*)) = 0.0703	*R*_1_ (*I* > 2σ(*I*)) = 0.0301; *wR*_2_ (*I* > 2σ(*I*)) = 0.0570
	*R*_1_ (all) = 0.0350; *wR*_2_ (all) = 0.0742	*R*_1_ (all) = 0.0382; *wR*_2_ (all) = 0.0585
largest diff. peak and hole	+6.124 e/Å^–3^ and −1.572 e/Å^–3^	+2.316 e/Å^–3^ and −2.165 e/Å^–3^
RMSD from mean	0.452 e/Å^–3^	0.353 e/Å^–3^
goodness-of-fit on *F*^2^	1.130	1.304

**Table 2:** Atomic Coordinates and Equivalent Isotropic Atomic Displacement Parameters (Å^2^) of La_3_Ni_2_O_7_-2222 at 300 K^a^

	Wyck.	*x*	*y*	*z*	occ.	*U*_eq_
La_1_	4*c*	0	0.75056(4)	1/4	1	0.00746(8)
La_2_	8*g*	0.32019(2)	0.25774(3)	1/4	1	0.00636(7)
Ni	8*g*	0.09588(3)	0.25241(6)	1/4	1	0.00470(10)
O_1_	8*e*	0.39563(15)	0	0	1	0.0113(5)
O_2_	8*e*	0.08964(15)	0	0	1	0.0111(5)
O_3_	8*g*	0.20433(15)	0.21690(6)	1/4	1	0.0131(5)
O_4_	4*c*	0	0.28950(8)	1/4	1	0.0114(6)

a *U*_eq_ is defined as one third of the trace of the orthogonalized *U*_*ij*_ tensor.

Furthermore, our observations reveal the existence of a hidden phase in this system, termed La_3_Ni_2_O_7_-1313, which adopts a strongly preferred *C*-centered orthorhombic lattice with the space group *Cmmm*. In its structural arrangement, the perovskite [LaNiO_3_] layers exhibit a systematic long-range order, alternating between single- and trilayer configurations (...1313...), as elucidated in Figure 1, represented by the chemical formula La_3_Ni_2_O_7_ being identical to that of bilayer La_3_Ni_2_O_7_-2222. The single crystal XRD refinement details of La_3_Ni_2_O_7_-1313 at 300 and 100 K are summarized in Tables 1 and 3, and Tables S1 and S2, respectively. Negligible vacancy has been observed regarding oxygen stoichiometry. Figure 1 also presents the characteristic stacking of single-, bilayer, and trilayer structures in Ruddlesden–Popper nickelates.^26,29^ A clear difference arises between La_3_Ni_2_O_7_-1313 and La_3_Ni_2_O_7_-2222 when considering perovskite layers and rock salt layers as the structural building blocks. The La_3_Ni_2_O_7_-1313 structure is constructed through a long-range order of single- and trilayer perovskite building blocks in La_2_NiO_4_ and La_4_Ni_3_O_10_, respectively, without the bilayer counterparts present in La_3_Ni_2_O_7_-2222. Recent research on the design and synthesis of a hybrid layered Ruddlesden–Popper nickelate by a flux method, featuring a ...1212... sequence,^30^ could provide a potential avenue for experimental validation.

**Table 3:** Atomic Coordinates and Equivalent Isotropic Atomic Displacement Parameters (Å^2^) of La_3_Ni_2_O_7_-1313 at 300 K^a^

	Wyck.	*x*	*y*	*z*	occ.	*U*_eq_
La_1_	4*l*	0	1/2	0.27343(2)	1	0.00909(8)
La_2_	4*l*	0	1/2	0.09348(2)	1	0.00953(8)
La_3_	4*k*	0	0	0.41317(2)	1	0.00914(8)
Ni_1_	4*k*	0	0	0.19063(4)	1	0.00627(13)
Ni_2_	2*c*	1/2	0	1/2	1	0.00779(19)
Ni_3_	2*a*	0	0	0	1	0.00550(18)
O_1_	4*l*	0	1/2	0.39120(3)	1	0.0300(17)
O_2_	4*f*	1/4	1/4	1/2	1	0.0132(9)
O_3_	4*k*	0	0	0.29700(3)	1	0.0277(16)
O_4_	8*m*	1/4	1/4	0.19070(2)	1	0.0179(8)
O_5_	4*k*	0	0	0.09380(3)	1	0.0341(18)
O_6_	4*e*	1/4	1/4	0	1	0.039(2)

a *U*_eq_ is defined as one third of the trace of the orthogonalized *U*_*ij*_ tensor.

Table 4 provides a summary of the Ni–O bond lengths and Ni–O–Ni bond angles in the structure of La_3_Ni_2_O_7_-1313. A noteworthy observation is the larger distortion of [NiO_6_] octahedra in the outer Ni of the trilayer (Ni1 in Figure 1) in comparison to the inner Ni of the trilayer (Ni3) and Ni in the single layer (Ni2). This aligns with reports in other multilayer Ruddlesden–Popper phases.^31^ Meanwhile, the Ni–O bond lengths within the perovskite layers on the outer side (Ni1–O3 and Ni2–O1) are approximately 10% greater than the Ni–O inside (Ni1–O5 and Ni3–O5) in La_3_Ni_2_O_7_-1313. To evaluate the valence states of three crystallographically unique Ni in the structure, bond valence sums were calculated (*R*_0_ = 1.689, *B* = 0.347).^32^ The obtained values for Ni in the single layer and inner and outer Ni in the trilayer reveal differences, indicating charge differentiation among them and providing evidence for potential charge transfer in the system. Remarkably, the bond valence sums of the inner and outer Ni in the trilayer of La_3_Ni_2_O_7_-1313 exhibit no significant difference from those reported in La_4_Ni_3_O_10_,^27^ suggesting a connection between trilayer building blocks.

**Table 4:** Selected Bond Lengths and Bond Angles in La_3_Ni_2_O_7_-1313^a^

	La_3_Ni_2_O_7_-1313
Ni–O (single layer)/Å	Ni2–O1: 2.211(6)
	Ni2–O2: 1.92674(5)
bond valence sum	2.46
Ni–O (outer Ni of the trilayer)/Å	Ni1–O3: 2.162(6)
	Ni1–O4: 1.92674(5)
	Ni1–O5: 1.969(6)
bond valence sums	2.72
Ni–O (inner Ni of the trilayer)/Å	Ni3–O5: 1.906(6)
	Ni3–O6: 1.92674(5)
bond valence sum	3.09
in-plane Ni–O–Ni/deg	Ni2–O2–Ni2: 180
	Ni1–O4–Ni1: 179.9(3)
	Ni3–O6–Ni3: 180
out-of-plane Ni–O–Ni/deg	Ni1–O5–Ni3: 180

a Crystallographically unique atoms are labeled in Figure 1.

**Figure 3:**
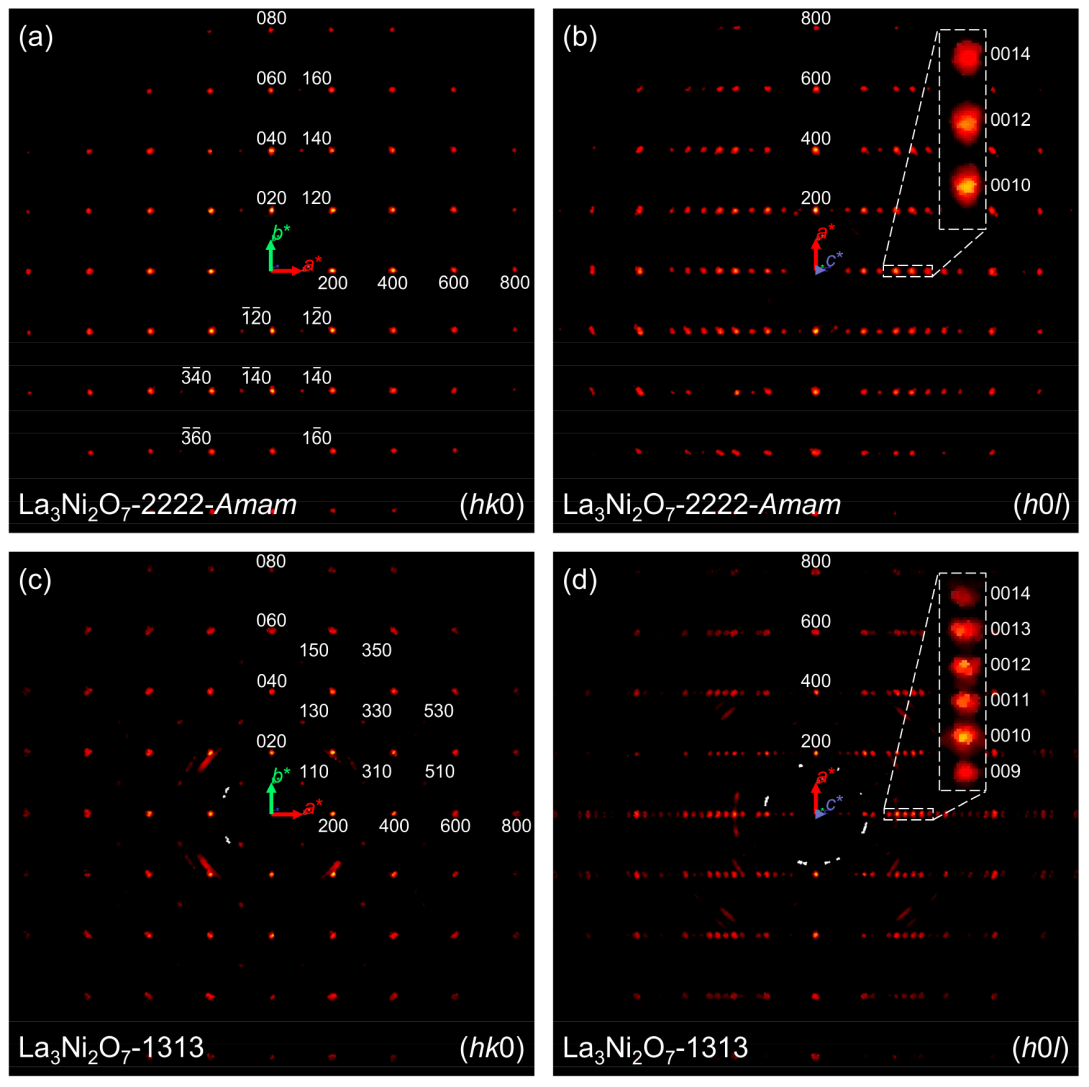
Reciprocal lattice planes of La_3_Ni_2_O_7_-1313 and La_3_Ni_2_O_7_-2222. (a, b) (*hk*0) and (*h*0*l*) planes of La_3_Ni_2_O_7_-2222. The *Cmcm* unit cell was transformed to an *Amam* setting for a better visual comparison with La_3_Ni_2_O_7_-1313. (c, d) (*hk*0) and (*h*0*l*) planes of La_3_Ni_2_O_7_-1313.

Our experimental reciprocal lattice planes are presented in Figure 3. To enable a clearer visual comparison between La_3_Ni_2_O_7_-1313 and La_3_Ni_2_O_7_-2222, the *Cmcm* unit cell (details in Table 1) of La_3_Ni_2_O_7_-2222 was transformed to a symmetry-equivalent *Amam* setting (*c* axis perpendicular to perovskite layers). Begin with La_3_Ni_2_O_7_-1313, given its space group *Cmmm*, and reflections at *h* + *k* = 2*n* would be expected due to *C*-centering, a condition met by all involved reflections. In contrast, for La_3_Ni_2_O_7_-2222, besides *A*-centering (*k* + *l* = 2*n*), the presence of the *a* glide plane perpendicular to the *b* axis results in absences in the (*h*0*l*) reciprocal plane when *h* = 2*n* + 1. Thus, the overall reflection conditions in the (*h*0*l*) plane are defined by *h* = 2*n* and *l* = 2*n*. Consequently, when examining the (*h*0*l*) plane, we expect to see twice as many reflections along c* when *h* = 2*n* in La_3_Ni_2_O_7_-1313 compared to La_3_Ni_2_O_7_-2222. Figure 3a and c provide a visual representation of the distinct reflection conditions due to lattice centering, while Figure 3b and d precisely illustrate the two distinct space groups in La_3_Ni_2_O_7_-2222 and La_3_Ni_2_O_7_-1313 as discussed above. Additionally, the (0*kl*) reciprocal planes in Figure S1 further validate the differences in the reflection conditions because of lattice centering.

However, we also noted weak reflections at half-integer positions within the current reciprocal lattice in the (0*kl*) plane in La_3_Ni_2_O_7_-1313, shown in Figure S2. This observation may suggest a lower symmetry or a mixture of *Cmmm* and the lower symmetry, as mentioned in a recent report on La_3_Ni_2_O_7_-1313.^33^ It is crucial to note that this observation does not compromise the distinctive ...1313... stacking sequence. The precise determination has been somewhat restricted by home lab single crystal XRD and may necessitate further examination through synchrotron X-ray and neutron diffraction.

### Electrical Resistivity Measurement

**Figure 4:**
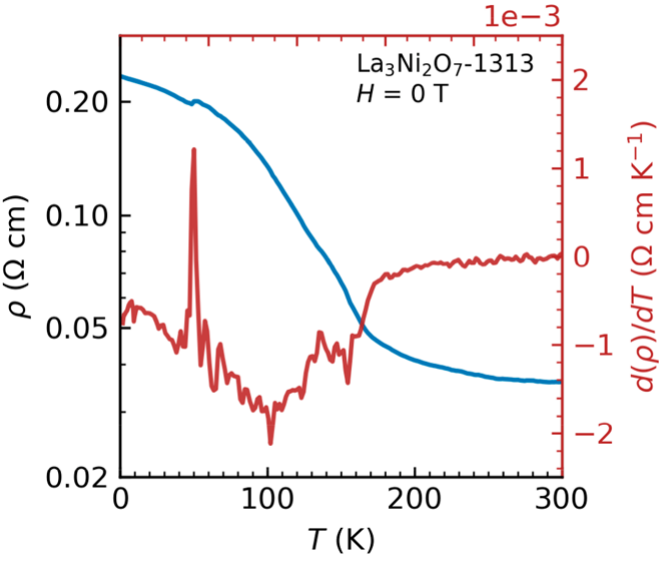
Temperature-dependent electrical resistivity and its temperature derivative of La_3_Ni_2_O_7_-1313. The kink at ∼50 K comes from unstable contacts.

The temperature dependence of electrical resistivity and its temperature derivative of La_3_Ni_2_O_7_-1313 in the range of 2–300 K was presented in Figure 4. The resistivity at 300 K measures approximately 0.035 Ω cm, a value small enough to preclude the presence of significant contact resistance. Our measurements indicate that the sample exhibit a semiconductor-like behavior, a departure from the characteristics reported for La_3_Ni_2_O_7_-2222.^17^ A kink at about 50 K was observed in our resistivity curve, which was attributed to unstable contacts. It is essential to emphasize that this kink should not be accounted for in the intrinsic properties of La_3_Ni_2_O_7_-1313 but rather considered as a result of contact instability. Two very recent reports on La_3_Ni_2_O_7_-1313 notes metallic behavior at ambient pressure,^33,34^ suggesting sample-dependent electrical resistivity. Considering the structure building blocks derived from Ruddlesden–Popper single layer La_2_NiO_4_ and trilayer La_4_Ni_3_O_10_ in La_3_Ni_2_O_7_-1313, we propose that the efficiency of charge-transfer between Ni in these single- and trilayer perovskite layers may dictate the bulk electrical resistivity behaviors of this system.

## Conclusion

In conclusion, we present the crystal structure and electrical resistivity of two Ruddlesden–Popper nickelates: bilayer La_3_Ni_2_O_7_-2222, and the hidden phase, La_3_Ni_2_O_7_-1313. Our single crystal XRD measurements confirm that La_3_Ni_2_O_7_-2222 features the pseudo *F*-centered orthorhombic lattice with bilayer perovskite [LaNiO_3_] layers separated by rock salt [LaO] layers (...2222...). Remarkably, the semiconductor-like La_3_Ni_2_O_7_-1313 adopts a strongly preferred *C*-centered orthorhombic lattice with space group *Cmmm*. In the structure, perovskite [LaNiO_3_] layers exhibit a systematic long-range order, alternating between single- and trilayer configurations (...1313...). This report introduces new possibilities for exploring the crystal structure and structure–property relationship in Ruddlesden–Popper nickelates.

## Supporting Information Available

Reciprocal lattice planes, (0*kl*), of La_3_Ni_2_O_7_-2222 and La_3_Ni_2_O_7_-1313; zoom-in (0*kl*) plane of La_3_Ni_2_O_7_-1313; crystal structure and refinement of La_3_Ni_2_O_7_-1313 at 100 K; atomic coordinates and equivalent isotropic atomic displacement parameters of La_3_Ni_2_O_7_-1313 at 100 K (PDF)
