# Supplementary material for: Long-Range Structural Order in a Hidden Phase of Ruddlesden–Popper Bilayer Nickelate La3Ni2O7
Source: Inorg Chem. 2024 Mar 5;63(11):5020–6. doi: 10.1021/acs.inorgchem.3c04474 (PMC10951943; doi:10.1021/acs.inorgchem.3c04474)
Supplement: Supplementary file 1 — ic3c04474_si_001.pdf [file ic3c04474_si_001.pdf]

Supporting Information

Long-Range Structural Order in a Hidden Phase of Ruddlesden–Popper Bilayer Nickelate $La_3Ni_2O_7$

Haozhe Wang[1], Long Chen[2], Aya Rutherford[2], Haidong Zhou[2], Weiwei Xie[1]*

1. Department of Chemistry, Michigan State University, East Lansing, MI, 48824, USA
2. Department of Physics and Astronomy, University of Tennessee, Knoxville, TN, 37996, USA

* Email: xieweiwe@msu.edu

Table of Contents

Figure S1 Reciprocal lattice planes, (0*kl*), of $La_3Ni_2O_7$-2222 and $La_3Ni_2O_7$-1313 ....S2
Figure S2 Zoom-in (0*kl*) plane of $La_3Ni_2O_7$-1313 ....S3

Table S1 The crystal structure and refinement of $La_3Ni_2O_7$-1313 at 100 K...S4
Table S2 Atomic coordinates and equivalent isotropic atomic displacement parameters of $La_3Ni_2O_7$-1313 at 100 K...S4

Figure S1 Reciprocal lattice planes, (0*kl*), of ***(a)*** $La_3Ni_2O_7$-2222 and ***(b)*** $La_3Ni_2O_7$-1313. At $k = 2n$, we observe twice as many reflections along c^*.

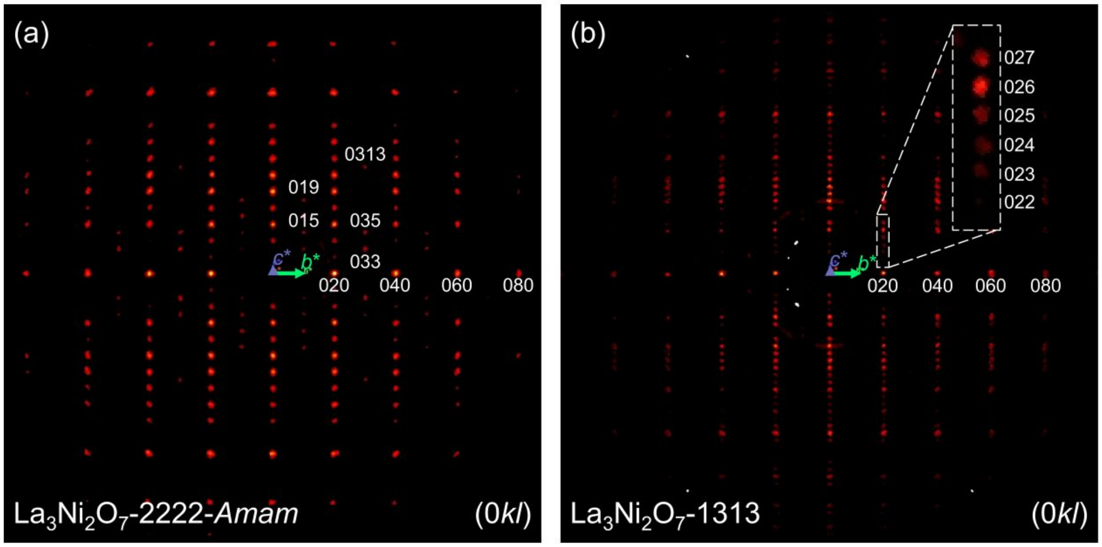

Figure S2 Zoom-in (0*kl*) plane of $La_3Ni_2O_7$-1313. Weak reflections at half-integer positions have been observed.

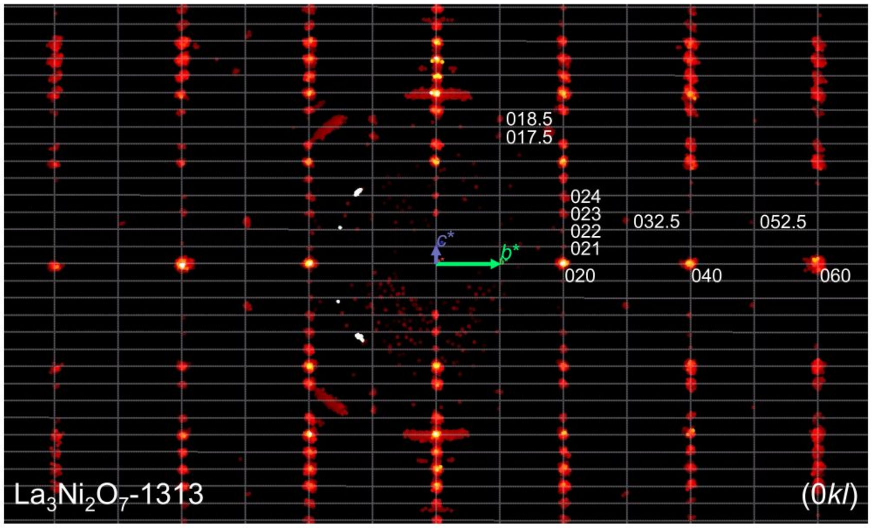

Table S1 The crystal structure and refinement of $La_3Ni_2O_7$-1313 at 100 K.

**Chemical Formula**	**$La_3Ni_2O_7$-1313**
Formula weight	646.15 g/mol
Space Group	*Cmmm*
Unit cell dimensions	a = 5.4265(2) Å<br>b = 5.4521(2) Å<br>c = 20.2969(6) Å
Volume	600.50(4) $Å^3$
Z	4
Density (calculated)	7.147 g/cm^3
Absorption coefficient	27.022 mm^{-1}
F(000)	1132
2θ range	6.02 to 81.48°
Reflections collected	14765
Independent reflections	1123 [R_{int} = 0.0568]
Refinement method	Full-matrix least-squares on F^2
Data / restraints / parameters	1123 / 0 / 48
Final R indices	R_1 (I>2σ(I)) = 0.0493; wR_2 (I>2σ(I)) = 0.0967<br>R_1 (all) = 0.0547; wR_2 (all) = 0.0982
Largest diff. peak and hole	+4.223 e/$Å^{-3}$ and –8.600 e/$Å^{-3}$
R.M.S. deviation from mean	0.543 e/$Å^{-3}$
Goodness-of-fit on F^2	1.439

Table S2 Atomic coordinates and equivalent isotropic atomic displacement parameters ($Å^2$) of $La_3Ni_2O_7$-1313 at 100 K. U_{eq} is defined as one third of the trace of the orthogonalized U_{ij} tensor.

	**Wyck.**	***x***	***y***	***z***	**Occ.**	**U_{eq}**
**La_1**	4*k*	0	0	0.22638(4)	1	0.00704(13)
**La_2**	4*l*	0	1/2	0.08680(3)	1	0.00684(12)
**La_3**	4*k*	0	0	0.40619(4)	1	0.00837(13)
**Ni_1**	4*l*	0	1/2	0.30907(8)	1	0.0054(3)
**Ni_2**	2*a*	0	0	0	1	0.0064(4)
**Ni_3**	2*c*	0	1/2	1/2	1	0.0048(3)
**O_1**	4*e*	1/4	1/4	0	1	0.0113(17)
**O_2**	8*m*	1/4	1/4	0.3093(4)	1	0.0156(14)
**O_3**	4*l*	0	1/2	0.2021(6)	1	0.019(2)
**O_4**	4*f*	1/4	1/4	1/2	1	0.046(5)
**O_5**	4*l*	0	1/2	0.4058(6)	1	0.036(4)
**O_6**	4*k*	0	0	0.1082(6)	1	0.029(3)
